# Synaptic connectivity amongst components of the locomotor central pattern generator

**DOI:** 10.3389/fncir.2022.1076766

**Published:** 2022-11-24

**Authors:** Simon Gosgnach

**Affiliations:** Department of Physiology, University of Alberta, Edmonton, AB, Canada

**Keywords:** interneuron, connectivity, CPG, locomotion, motor control

## Abstract

In the past two decades we have learned an enormous amount of information regarding the identity of functional components of the neural circuitry responsible for generating locomotor activity in mammals. Molecular techniques, combined with classic electrophysiological and anatomical approaches, have resulted in the identification of a handful of classes of genetically defined interneuronal populations, and a delineation of the specific function of many of these during stepping. What lags behind at this point is a clear picture of the synaptic connectivity of each population, this information is key if we are to understand how the interneuronal components that are responsible for locomotor activity work together to form a functional circuit. In this mini review I will summarize what is, and what is not, known regarding the synaptic connectivity of each genetically defined interneuronal population that is involved in locomotion.

## Introduction

Locomotion is an essential motor behavior carried out by all vertebrates. The alternation of flexor and extensor muscles on the left and right sides of the body, which is the hallmark of locomotor activity in most limbed mammals, relies on the activation of a neural circuit, situated in the spinal cord, commonly referred to as the locomotor central pattern generator (CPG, see Kiehn, 2016 for review). Given the fact that injury to the mammalian spinal cord is irreparable, and leads to the loss of sensorimotor function below the level of the lesion, the initial experiments demonstrating that the caudal spinal cord was capable of generating stepping movements (Brown, 1911) led to a great deal of experimental work focused on the identification and characterization of neuronal components of the locomotor CPG. Generally speaking, progress was made in determining the location of this neural network, and its pharmacological basis (Jankowska et al., 1967; Kjaerulff and Kiehn, 1996; Cowley and Schmidt, 1997), however, identification and characterization of the interneuronal components of the locomotor CPG proved extraordinarily challenging. This was largely due to the vast number of neurons in the spinal cord, and the extent to which neurons with similar properties and features are intermingled with other, unrelated neurons.

Towards the end of the 20th century work primarily spearheaded by the labs of Tom Jessel and Martyn Goulding incorporated a molecular approach to overcome these obstacles (Bang and Goulding, 1996; Tanabe and Jessell, 1996). Interneurons in the ventral spinal cord were divided up into a handful of genetically distinct populations, and analysis indicated that each shared many intrinsic properties, suggesting that they may represent functionally homogeneous populations that play a specific role during locomotor activity (Grillner and Jessell, 2009). In the ventral spinal cord, the region in which the primary components of the locomotor CPG are situated, a handful of populations have been shown to be involved in locomotor activity (Goulding et al., 2002). Subsequent genetic silencing or ablation of each population has enabled elucidation of the specific function of each during locomotor activity (see Goulding, 2009; Kiehn, 2016 for review). As a result of this work we now have an excellent grasp of the specific neurons responsible for such key functions as left-right alternation, flexor-extensor alternation, locomotor stability, and modulation of locomotor speed. Since it is possible to label these populations with reporter proteins it is also possible to visualize them, and target each directly for further investigation.

In order to understand how each of these genetically defined interneuronal components work together to form a functional network it is necessary to map the synaptic connectivity of each. While a myriad of anterograde and retrograde tracing techniques are available (Callaway, 2005), construction of a “network map” of the locomotor CPG has proven challenging for a couple of reasons. First of all, not all members of a given interneuronal population are locomotor- related. Whole cell recordings have not been made from many of the genetically defined interneuronal populations and, in those that have been analyzed, as little as half of a given population has been shown to be rhythmically active and locomotor-related during stepping (Dougherty and Kiehn, 2010; Dougherty et al., 2013). Thus, even if synaptic connectivity of a given population is revealed, it is difficult to determine whether the connections identified are from the subset that are locomotor- related. Second, neurons have numerous axon collaterals, so while it is relatively easy to ask whether members of a given population project to a certain region or cell type (i.e., do V0 cells contact motoneurons), it is much more difficult to determine all of the synaptic partners of a population (i.e., to which cell types do V0 cells project).

In this mini review I will focus on each of the genetically defined spinal interneuron populations in the ventral spinal cord that have thus far been identified to play a role during locomotor activity in limbed mammals, and assess what has been demonstrated experimentally regarding their synaptic connectivity. One of the primary goals of this mini review is to distinguish what has been shown experimentally as opposed to what has been hypothesized. I will therefore not discuss valuable findings from experiments carried out in other species, or computational modeling studies. Taking into account the conclusions using each of these approaches will inform future studies directed at describing the network function of the mammalian locomotor CPG.

## V0 interneurons

V0 interneurons were the first of the parent genetically defined classes to be characterized, and have their functional role in stepping determined (Lanuza et al., 2004). Initial work demonstrated that this population expresses the transcription factor *Dbx1*, and application of fluorescent dextran to one side of the developing spinal cord indicated that approximately 95% of this population project contralateral axons that cross the midline at the ventral commissure (Moran-Rivard et al., 2001; Pierani et al., 2001). Subsequent study revealed the role of these cells in locomotor activity by demonstrating that in the absence of *Dbx1*-expressing neurons newborn mice have severe issues with left/right alternation (Lanuza et al., 2004). This study looked more closely at the synaptic connectivity of V0 cells and analyzed the transport of the retrograde transynaptic tracer pseudorabies virus (PRV-152) that had been applied to hindlimb muscles to determine whether V0 cells contact contralateral hindlimb motoneurons. While results indicated that they clearly do, no further investigations have been carried out to determine if this population has additional synaptic partners, a distinct possibility since there is no information regarding the proportion of V0 cells which project to motoneurons. An additional study demonstrated that the two primary subpopulations of *Dbx1* expressing cells (V0_V_ cells which also express the transcription factor *Evx1*, and V0_D_ cells which do not) have complimentary roles in locomotor activity, with V0_V_ cells responsible for left/right alternation at faster locomotor speeds while V0_D_ cells are responsible for this function during slower locomotion (Talpalar et al., 2013). In this study there is no additional information regarding the specific connectivity of either the V0_V_ or V0_D_ subtypes, however, it is extremely unlikely that both subsets project exclusively to contralateral motoneurons since they are both involved in left/right alternation and one of these subsets is excitatory (V0_V_) while the other is inhibitory (V0_D_).

Further analysis of the V0 population revealed that there is also a small subset of cholinergic interneurons (V0_C_ cells) which express the transcriptions factor *Pitx2* and modulate hindlimb motoneuron/muscle activity (Zagoraiou et al., 2009). These cells are cholinergic and comprise some, if not all, of the small number of V0 cells initially shown to project ipsilaterally (Moran-Rivard et al., 2001; Pierani et al., 2001). Analysis of synaptic terminals from V0_C_ neurons across the spinal cord indicate extensive connectivity onto motoneurons, while sparse synaptic boutons from this subpopulation of V0 neurons were also seen on interneurons in both the intermediate nucleus and ventral horn (Zagoraiou et al., 2009).

## V1 interneurons

V1 interneurons express the transcription factor *En1* and, as a whole, more than 90% of this population has been shown to be inhibitory (Sapir et al., 2004). Studies in which V1 neurons are either absent or silenced indicate that these cells are involved in the regulation of locomotor speed (Gosgnach et al., 2006), and they also work together with a subset of the V2 population to secure ipsilateral alternation of flexor and extensor motoneurons (Zhang et al., 2014; Britz et al., 2015). V1 neurons were initially shown to extend their axons exclusively on the ipsilateral side of the spinal cord (Saueressig et al., 1999), and it was later demonstrated that the physiologically characterized Renshaw cells (Sapir et al., 2004), and Ia inhibitory interneurons (Alvarez et al., 2005; Siembab et al., 2010; Benito-Gonzalez and Alvarez, 2012) are derived from this population. It is thus unsurprising that these studies demonstrated that synaptic terminals from V1 neurons appear on motoneurons, Renshaw cells, and Ia inhibitory interneurons (Alvarez et al., 2005; Siembab et al., 2010; Benito-Gonzalez and Alvarez, 2012; Bikoff et al., 2016). Further analysis of the specific connectivity on motoneurons demonstrated that synaptic terminals of V1 neurons preferentially contact flexor (as opposed to extensor) motor pools (Britz et al., 2015).

## V2 and Shox2 interneurons

The V2 interneuronal population can be divided up into two distinct subpopulations, excitatory V2a neurons which express the transcription factor *Chx10* (Al-Mosawie et al., 2007; Lundfald et al., 2007), and inhibitory V2b cells which express *Gata3* (Lundfald et al., 2007; Zhang et al., 2014; Britz et al., 2015). Initial characterization of the axonal projections of both subpopulations *via* fluorescent dextran application to the developing spinal cord indicated that all V2a neurons, and greater than 90% of V2b cells extend ipsilateral axons (Lundfald et al., 2007). V2a neurons are one of the few populations that have been electrophysiologically characterized *via* whole cell recordings during locomotor activity, and approximately 40% of this population has been shown to be rhythmically active and locomotor- related (Dougherty and Kiehn, 2010; Zhong et al., 2010). During these experiments the tracer biocytin was included with the intracellular solution in order to map the axonal projection of V2a neurons. Although no distinction was made between those V2a that were locomotor- related, and those that were not, axon terminals from V2a cells were found in the vicinity of commissural interneurons in the intermediate laminae of the spinal cord (Crone et al., 2008; Dougherty and Kiehn, 2010), and in the ventral horn, immediately surrounding motoneurons (Dougherty and Kiehn, 2010). The connectivity onto commissural interneurons fits nicely with the defects observed in left right alternation when the V2a neurons are ablated (Crone et al., 2008; Crone et al., 2009) as it is possible that the V0 population, which have been shown to be responsible for this alternation, rely on excitation from ipsilateral V2a neurons to execute their function.

The V2b subset has not been studied in nearly as much detail as the V2a cells, however, they have been shown to work together with V1 interneurons to coordinate flexor-extensor alternation (Zhang et al., 2014; Britz et al., 2015). Synaptic contacts from V2b neurons have been found primarily on extensor motoneurons (Zhang et al., 2014; Britz et al., 2015) although V2b neurons have also been found to terminate on V0_C_ cells as well as unidentified interneurons in lamina VII/VIII (Zhang et al., 2014).

The Shox2 + population does not fit neatly into the original genetically defined parent populations, however, approximately 75% of neurons expressing this transcription factor co-express Chx10, and thus belong to the V2a population (Dougherty et al., 2013). The 25% that do not (i.e., those that are Shox2 + and Chx10-) have been linked to the generation of locomotor activity in the spinal cord. Shox2 expressing cells are almost certain to have a significant role during locomotion as 67% of recorded neurons showed either membrane potential oscillations and/or spiking activity in phase with fictive locomotion in the neonatal spinal cord when whole cell recordings were made (Dougherty et al., 2013). Unfortunately, the inability to distinguish between the Chx10 + and Chx10- subpopulations of Shox2 neurons in live tissue has made it challenging to characterize those cells that express Shox2 alone and are likely to be involved in locomotor rhythmogenesis. Dextran backfills indicate that Shox2 cells project axons exclusively ipsilaterally (Dougherty et al., 2013), and intracellular fills of Shox2 neurons demonstrate that they contact motoneurons. Again, it is important to keep in mind that for these, and all anterograde tracing experiments it was not possible to distinguish between those Shox2 interneurons that did, and did not, express Chx10. Paired whole cell recording was used to demonstrate that Shox2 + expressing cells contact commissural interneurons (Dougherty et al., 2013) as well as other Shox2 neurons (Dougherty et al., 2013; Ha and Dougherty, 2018). Based on the fact that Chx10 expressing Shox2 neurons are situated more laterally in the spinal cord, and those Shox2 + cells that contact ipsilateral motoneurons are situated relatively laterally compared to those that do not, it has been suggested that the Shox2 + /Chx10 + subpopulation projects to motoneurons, while those cells that express Shox2 alone contact one another, or commissural interneurons and are likely to be involved in locomotor ryhthm generation, however, given that it has not been possible to visualize only those members of this population that are Shox2 + /Chx10- in live tissue and thus this has yet to be backed up with experimental data.

## V3 interneurons

The V3 population is excitatory and was initially shown to extend contralaterally projecting axons at early embryonic time points (Zhang et al., 2008). More recent work in older animals indicates that V3’s are a mixed population of ipsilaterally, and contralaterally, projecting neurons (Chopek et al., 2018). Ablation or silencing of the entire V3 population during locomotor activity results in a rather nuanced loss of coordination and regularity between the left and right hindlimbs, indicative of a role either in a variety of functions, or possibly a role in locomotor stability (Zhang et al., 2008). Earlier work with the retrograde transynaptic tracer PRV-152 demonstrated that this population contacted contralateral motoneurons, and investigation of synaptic terminals of labeled V3 cells indicated that they also terminate on Renshaw cells and Ia inhibitory interneurons (Zhang et al., 2008).

More recent work has divided up the ventrally located V3 population into a medial (V3_VMed_) and lateral (V3_VLat_) subset based on their location in the spinal cord, and an investigation of each has revealed an interesting connectivity pattern, including extensive ipsilateral synaptic contacts (Chopek et al., 2018). V3_VLat_ neurons were optogenetically activated and shown to evoke monosynaptic responses in motoneurons situated ipsilaterally, while activation of V3_VMed_ cells evoked monosynaptic responses in both ipsilateral V3_VMed_ and V3_VLat_ neurons (Chopek et al., 2018). Retrograde labeling of both subsets of ventrally located V3 neurons indicated that, in addition to these ipsilaterally projecting axons, many bifurcate and also project axons contralaterally. Importantly, the activity of V3 neurons have yet to be assessed during locomotor activity, and we thus have no knowledge of the proportion of this diverse population that is locomotor-related.

## Discussion

In this mini review I have provided an overview of the experimental findings regarding synaptic connectivity of the ventrally situated interneuronal populations shown to be involved in locomotor activity. I have not included data generated from computational modeling work here. This work is of enormous importance as it provides predictions regarding the detailed synaptic connectivity that is able to account for coordinated locomotion (Rybak et al., 2015; Shevtsova et al., 2015; Shevtsova and Rybak, 2016; Danner et al., 2019; Shevtsova et al., 2020; Shevtsova et al., 2022). It can thus lead directly to testable hypotheses and the design of experiments which investigate whether the predictions are accurate, however, it should not be taken as fact until tracing experiments prove it to be so. I have also left out information regarding connectivity amongst interneurons that comprise the locomotor CPG in the “simpler” nervous system of non-mammalian species. Partially due to their more accessible nervous system, more progress has been made unraveling the connectivity of the locomotor circuitry in these species, however, in many cases the function of the genetically defined interneuronal populations is different than that in limbed mammals (reviewed in Buschges et al., 2011; Grillner and El Manira, 2015; Berg et al., 2018). As with the computational modeling experiments, findings in these species are extremely valuable as they can lead to experiments aimed at determining whether the connectivity is maintained across species, however, caution must be exhibited when translating these findings to limbed mammals.

Given the data collected over the past two decades what is our current knowledge regarding the synaptic connectivity of genetically defined components of the mammalian locomotor CPG? At this point, other than the V0 interneurons, each of the ventrally situated parent populations, and subpopulations, have had their synaptic terminals assessed and have been shown to project to multiple identified neuronal groups (see Table 1). The V0 neurons have been shown to project to contralateral motoneurons, however, this was determined using a “closed ended” approach in which only those contacts onto motoneurons would be revealed. These experiments leave us unclear on the proportion of V0 cells which project to motoneurons, and also provide no information regarding other synaptic partners of this population.

**TABLE 1:** Summary of what is known, and some of the knowledge is lacking, regarding the synaptic connectivity of each ventrally-located, interneuronal population involved in locomotor activity.

Cell type	Direction of axonal projection	Tracing assay used	Identified synaptic partner(s)	% of population loco-related
V0_D_	Contralateral	Retrograde PRV tracing from MNs	MNs	Unknown
V0_V_				
V0_C_	Ipsilateral	Anterograde analysis of synaptic terminals	MNs	Unknown

V1	Ipsilateral	Anterograde analysis of synaptic terminals	Flexor MNs/V1 INs	Unknown

V2a	Ipsilateral	Anterograde biocytin fills	Commissural INs and MNs	Approx. 40%

V2b	> 90% Ipsilateral	Anterograde analysis of synaptic terminals	Extensor MNs/laminae Vii/VIII INs, V0_C_ neurons	Unknown

V3_VLat_	Ipsilateral and Contralateral	Retrograde PRV tracing from MNs. Monosynaptic electrophysiological recording.	Ipsilateral/Contralateral MNS	Unknown
V3_VMed_	Ipsilateral and Contralateral		V3_VLat_, V3_VMed_ populations	Unknown

Shox2	Ipsilateral	Monosynaptic electrophysiological recording. Anterograde, analysis of synaptic terminals	Other Shox2 neurons, MNs, commissural INs	Approx 67%

Even with all of the data collected on each interneuronal population, a major factor that is hindering the construction of a comprehensive network map of the locomotor CPG in mammals is the inability to study the axonal projections of only those neurons belonging to each population that are locomotor- related. Thus far only the V2a, and Shox2 + cells (a population which will have included many V2a neurons) have been recorded from during locomotor-like activity. These experiments have shown that between 40 and 70% of neurons are rhythmically active and either in phase, or out of phase, with rhythmic, locomotor-like activity recorded from the ventral roots (Dougherty and Kiehn, 2010; Dougherty et al., 2013). Since axonal tracing experiments, including those that have analyzed connectivity of the V2a and Shox2 + neurons, have not taken into account whether the cells analyzed are rhythmically active during locomotor activity, interpretation of the results must be taken with a grain of salt as we have no way of knowing whether the neurons, and terminals, studied are even active participants during stepping. Complicating things even further is the modular nature of the locomotor CPG with different populations active at certain locomotor frequencies but presumably silent at other frequencies (Hagglund et al., 2013; Rancic et al., 2020).

None of this should come as a surprise, mapping connectivity amongst neurons in a complex network is technically difficult, and in the case of the locomotor CPG this is further complicated by the fact that the spinal cord must remain largely intact in order for the network to operate. Perhaps the best approach is to use semi intact (Dyck and Gosgnach, 2009; Dougherty et al., 2013) or imaging approaches (Rancic et al., 2019) to first identify genetically labeled populations that are rhythmically active during locomotor-like activity and then fill the axons of only those cells that are locomotor related. Undoubtably these experiments would be tedious, but potentially required to make some sense of connectivity amongst neurons that comprise the black box which is the mammalian locomotor CPG.

## Author contributions

SG wrote the manuscript, contributed to the article and approved the submitted version.

## References

[B1] Al-Mosawie A. Wilson J. M. Brownstone R. M. (2007). Heterogeneity of V2-derived interneurons in the adult mouse spinal cord. *Eur. J. Neurosci.* 26 3003–3015. 10.1111/j.1460-9568.2007.05907.x 18028108

[B2] Alvarez F. J. Jonas P. C. Sapir T. Hartley R. Berrocal M. C. Geiman E. J. (2005). Postnatal phenotype and localization of spinal cord V1 derived interneurons. *J. Comp. Neurol.* 493 177–192. 10.1002/cne.20711 16255029 PMC2997483

[B3] Bang A. G. Goulding M. D. (1996). Regulation of vertebrate neural cell fate by transcription factors. *Curr. Opin. Neurobiol.* 6 25–32. 8794048 10.1016/s0959-4388(96)80005-5

[B4] Benito-Gonzalez A. Alvarez F. J. (2012). Renshaw cells and Ia inhibitory interneurons are generated at different times from p1 progenitors and differentiate shortly after exiting the cell cycle. *J. Neurosci.* 32 1156–1170. 10.1523/JNEUROSCI.3630-12.2012 22279202 PMC3276112

[B5] Berg E. M. Björnfors E. R. Pallucchi I. Picton L. D. El Manira A. (2018). Principles governing locomotion in vertebrates: Lessons from zebrafish. *Front. Neural. Circuits.* 12:73. 10.3389/fncir.2018.00073 30271327 PMC6146226

[B6] Bikoff J. B. Gabitto M. I. Rivard A. F. Drobac E. Machado T. A. Miri A. (2016). Spinal inhibitory interneuron diversity delineates variant motor microcircuits. *Cell* 165 207–219. 10.1016/j.cell.2016.01.027 26949184 PMC4808435

[B7] Britz O. Zhang J. Grossmann K. S. Dyck J. Kim J. C. Dymecki S. (2015). A genetically defined asymmetry underlies the inhibitory control of flexor-extensor locomotor movements. *eLife* 4:e04718. 26465208 10.7554/eLife.04718 PMC4604447

[B8] Brown T. G. (1911). The intrinsic factors in the act of progression in mammals. *Proc. R. Soc. B* 84 308–319.

[B9] Buschges A. Scholz H. El Manira A. (2011). New moves in motor control. *Curr. Biol.* 21 R513–R524. 21741590 10.1016/j.cub.2011.05.029

[B10] Callaway E. M. (2005). A molecular and genetic arsenal for systems neuroscience. *Trends Neurosci.* 28 196–201. 15808354 10.1016/j.tins.2005.01.007

[B11] Chopek J. W. Nascimento F. Beato M. Brownstone R. M. Zhang Y. (2018). Sub-populations of spinal V3 interneurons form focal modules of layered pre-motor microcircuits. *Cell Rep.* 25 146–156. 10.1016/j.celrep.2018.08.095 30282024 PMC6180347

[B12] Cowley K. C. Schmidt B. J. (1997). Regional distribution of the locomotor pattern-generating network in the neonatal rat spinal cord. *J. Neurophysiol.* 77 247–259. 10.1152/jn.1997.77.1.247 9120567

[B13] Crone S. A. Quinlan K. A. Zagoraiou L. Droho S. Restrepo C. E. Lundfald L. (2008). Genetic ablation of V2a ipsilateral interneurons disrupts left-right locomotor coordination in mammalian spinal cord. *Neuron* 60 70–83. 10.1016/j.neuron.2008.08.009 18940589

[B14] Crone S. A. Zhong G. Harris-Warrick R. Sharma K. (2009). In mice lacking V2a interneurons, gait depends on speed of locomotion. *J. Neurosci.* 29 7098–7109. 19474336 10.1523/JNEUROSCI.1206-09.2009 PMC2731420

[B15] Danner S. M. Zhang H. Shevtsova N. A. Borowska-Fielding J. Deska-Gauthier D. Rybak I. A. (2019). Spinal V3 interneurons and left-right coordination in mammalian locomotion. *Front. Cell Neurosci.* 13:516. 10.3389/fncel.2019.00516 31824266 PMC6879559

[B16] Dougherty K. J. Kiehn O. (2010). Functional organization of V2a-related locomotor circuits in the rodent spinal cord. *Ann. N. Y. Acad. Sci.* 1198 85–93. 10.1111/j.1749-6632.2010.05502.x 20536923

[B17] Dougherty K. J. Zagoraiou L. Satoh D. Rozani I. Doobar S. Arber S. (2013). Locomotor rhythm generation linked to the output of spinal shox2 excitatory interneurons. *Neuron* 80 920–933. 10.1016/j.neuron.2013.08.015 24267650

[B18] Dyck J. Gosgnach S. (2009). Whole cell recordings from visualized neurons in the inner laminae of the functionally intact spinal cord. *J. Neurophysiol.* 102 590–597. 10.1152/jn.00212.2009 19386756

[B19] Gosgnach S. Lanuza G. M. Butt S. J. Saueressig H. Zhang Y. Velasquez T. (2006). V1 spinal neurons regulate the speed of vertebrate locomotor outputs. *Nature* 440 215–219. 10.1038/nature04545 16525473

[B20] Goulding M. (2009). Circuits controlling vertebrate locomotion: Moving in a new direction. *Nat. Rev. Neurosci.* 10 507–518. 10.1038/nrn2608 19543221 PMC2847453

[B21] Goulding M. Lanuza G. Sapir T. Narayan S. (2002). The formation of sensorimotor circuits. *Curr. Opin. Neurobiol.* 12 508–515. 12367629 10.1016/s0959-4388(02)00371-9

[B22] Grillner S. El Manira A. (2015). The intrinsic operation of the networks that make us locomote. *Curr. Opin. Neurobiol.* 31 244–249. 25599926 10.1016/j.conb.2015.01.003

[B23] Grillner S. Jessell T. M. (2009). Measured motion: Searching for simplicity in spinal locomotor networks. *Curr. Opin. Neurobiol.* 19 572–586. 10.1016/j.conb.2009.10.011 19896834 PMC2951840

[B24] Ha N. T. Dougherty K. J. (2018). Spinal Shox2 interneuron interconnectivity related to function and development. *eLife* 7:e42519. 30596374 10.7554/eLife.42519 PMC6333440

[B25] Hagglund M. Dougherty K. J. Borgius L. Itohara S. Iwasato T. Kiehn O. (2013). Optogenetic dissection reveals multiple rhythmogenic modules underlying locomotion. *Proc. Natl. Acad. Sci. U.S.A.* 110 11589–11594. 10.1073/pnas.1304365110 23798384 PMC3710792

[B26] Jankowska E. Jukes M. G. M. Lund S. Lundberg A. (1967). The effect of DOPA on the spinal cord. 6. Half-centre organization of interneurones transmitting effects from the flexor reflex afferents. *Acta Physiol. Scand.* 70 389–403. 10.1111/j.1748-1716.1967.tb03637.x 4294400

[B27] Kiehn O. (2016). Decoding the organization of spinal circuits that control locomotion. *Nat. Rev. Neurosci.* 17 224–238. 26935168 10.1038/nrn.2016.9 PMC4844028

[B28] Kjaerulff O. Kiehn O. (1996). Distribution of networks generating and coordinating locomotor activity in the neonatal rat spinal cord in vitro: A lesion study. *J. Neurosci.* 16 5777–5794. 10.1523/JNEUROSCI.16-18-05777.1996 8795632 PMC6578971

[B29] Lanuza G. M. Gosgnach S. Pierani A. Jessell T. M. Goulding M. (2004). Genetic identification of spinal interneurons that coordinate left-right locomotor activity necessary for walking movements. *Neuron* 42 375–386. 10.1016/s0896-6273(04)00249-1 15134635

[B30] Lundfald L. Restrepo C. E. Butt S. J. Peng C. Y. Droho S. Endo T. (2007). Phenotype of V2-derived interneurons and their relationship to the axon guidance molecule EphA4 in the developing mouse spinal cord. *Eur. J. Neurosci.* 26 2989–3002. 10.1111/j.1460-9568.2007.05906.x 18028107

[B31] Moran-Rivard L. Kagawa T. Saueressig H. Gross M. K. Burrill J. Goulding M. (2001). Evx1 is a postmitotic determinant of v0 interneuron identity in the spinal cord. *Neuron* 29 385–399. 10.1016/s0896-6273(01)00213-6 11239430

[B32] Pierani A. Moran-Rivard L. Sunshine M. J. Littman D. R. Goulding M. Jessell T. M. (2001). Control of interneuron fate in the developing spinal cord by the progenitor homeodomain protein Dbx1. *Neuron* 29 367–384. 11239429 10.1016/s0896-6273(01)00212-4

[B33] Rancic V. Ballanyi K. Gosgnach S. (2020). Mapping the dynamic recruitment of spinal neurons during fictive locomotion. *J. Neurosci.* 40 9692–9700. 10.1523/JNEUROSCI.1885-20.2020 33188068 PMC7726538

[B34] Rancic V. Haque F. Ballanyi K. Gosgnach S. (2019). Using an upright preparation to identify and characterize locomotor related neurons across the transverse plane of the neonatal mouse spinal cord. *J. Neurosci. Methods.* 323, 90–97. 10.1016/j.jneumeth.2019.05.010 31132372

[B35] Rybak I. A. Dougherty K. J. Shevtsova N. A. (2015). Organization of the mammalian locomotor CPG: Review of computational model and circuit architectures based on genetically identified spinal interneurons(1,2,3). *eNeuro* 2 ENEURO.0069-15.2015. 10.1523/ENEURO.0069-15.2015 26478909 PMC4603253

[B36] Sapir T. Geiman E. J. Wang Z. Velasquez T. Mitsui S. Yoshihara Y. (2004). Pax6 and engrailed 1 regulate two distinct aspects of renshaw cell development. *J. Neurosci.* 24 1255–1264. 10.1523/JNEUROSCI.3187-03.2004 14762144 PMC2997484

[B37] Saueressig H. Burrill J. Goulding M. (1999). Engrailed-1 and netrin-1 regulate axon pathfinding by association interneurons that project to motor neurons. *Development* 126 4201–4212. 10.1242/dev.126.19.4201 10477289

[B38] Shevtsova N. A. Rybak I. A. (2016). Organization of flexor-extensor interactions in the mammalian spinal cord: Insights from computational modelling. *J. Physiol.* 594 6117–6131. 10.1113/JP272437 27292055 PMC5088238

[B39] Shevtsova N. A. Ha N. T. Rybak I. A. Dougherty K. J. (2020). Neural Interactions in developing rhythmogenic spinal networks: Insights from computational modeling. *Front. Neural Circuits.* 14:614615. 10.3389/fncir.2020.614615 33424558 PMC7787004

[B40] Shevtsova N. A. Li E. Z. Singh S. Dougherty K. J. Rybak I. A. (2022). Ipsilateral and contralateral interactions in spinal locomotor circuits mediated by V1 neurons: Insights from computational modeling. *Int. J. Mol. Sci.* 23:5541. 10.3390/ijms23105541 35628347 PMC9146873

[B41] Shevtsova N. A. Talpalar A. E. Markin S. N. Harris-Warrick R. M. Kiehn O. Rybak I. A. (2015). Organization of left-right coordination of neuronal activity in the mammalian spinal cord: Insights from computational modelling. *J. Physiol.* 2015 2403–2426. 10.1113/JP270121 PMC4461406 25820677

[B42] Siembab V. C. Smith C. A. Zagoraiou L. Berrocal M. C. Mentis G. Z. Alvarez F. J. (2010). Target selection of proprioceptive and motor axon synapses on neonatal V1-derived Ia inhibitory interneurons and Renshaw cells. *J. Comp. Neurol.* 518 4675–3701. 10.1002/cne.22441 20963823 PMC3038681

[B43] Talpalar A. E. Bouvier J. Borgius L. Fortin G. Pierani A. Kiehn O. (2013). Dual-mode operation of neuronal networks involved in left-right alternation. *Nature* 500 85–88. 10.1038/nature12286 23812590

[B44] Tanabe Y. Jessell T. M. (1996). Diversity and pattern in the developing spinal cord. *Science* 274 1115–1123. 8895454 10.1126/science.274.5290.1115

[B45] Zagoraiou L. Akay T. Martin J. F. Brownstone R. M. Jessell T. M. Miles G. B. (2009). A cluster of cholinergic premotor interneurons modulates mouse locomotor activity. *Neuron* 64 645–662. 20005822 10.1016/j.neuron.2009.10.017 PMC2891428

[B46] Zhang J. Lanuza G. M. Britz O. Wang Z. Siembab V. C. Zhang Y. (2014). V1 and v2b interneurons secure the alternating flexor-extensor motor activity mice require for limbed locomotion. *Neuron* 82 138–150. 10.1016/j.neuron.2014.02.013 24698273 PMC4096991

[B47] Zhang Y. Narayan S. Geiman E. Lanuza G. M. Velasquez T. Shanks B. (2008). V3 spinal neurons establish a robust and balanced locomotor rhythm during walking. *Neuron* 60 84–96. 10.1016/j.neuron.2008.09.027 18940590 PMC2753604

[B48] Zhong G. Droho S. Crone S. A. Dietz S. Kwan A. C. Webb W. W. (2010). Electrophysiological characterization of V2a interneurons and their locomotor-related activity in the neonatal mouse spinal cord. *J. Neurosci.* 30 170–182. 10.1523/JNEUROSCI.4849-09.2010 20053899 PMC2824326

